# In Vitro Propagation of the Blueberry ‘Blue Suede™’ (*Vaccinium* hybrid) in Semi-Solid Medium and Temporary Immersion Bioreactors

**DOI:** 10.3390/plants12152752

**Published:** 2023-07-25

**Authors:** Kim-Cuong Le, Shannon Johnson, Cyrus K. Aidun, Ulrika Egertsdotter

**Affiliations:** 1 G.W. Woodruff School of Mechanical Engineering, Georgia Institute of Technology, 500 10th Street NW, Atlanta, GA 30332-0620, USA; cuong.le@me.gatech.edu (K.-C.L.); shannon.johnson@me.gatech.edu (S.J.); cyrus.aidun@me.gatech.edu (C.K.A.); 2 Renewable Bioproducts Institute, Georgia Institute of Technology, 500 10th Street NW, Atlanta, GA 30332-0620, USA; 3 Department of Forest Genetics and Plant Physiology, Umea Plant Science Center (UPSC), Swedish University of Agricultural Science (SLU), 901-83 Umea, Sweden

**Keywords:** blueberry, ‘Blue Suede™’ (*Vaccinium* hybrid), micropropagation, temporary immersion bioreactor

## Abstract

The production of blueberries for fresh and processed consumption is increasing globally and has more than doubled in the last decade. Blueberry is grown commercially across a variety of climates in over 30 countries. The major classes of plants utilized for the planting and breeding of new cultivars are highbush, lowbush, half-high, Rabbiteye, and Southern highbush. Plants can be propagated by cuttings or in vitro micropropagation techniques. In vitro propagation offers advantages for faster generation of a large number of disease-free plants independent of season. Labor costs for in vitro propagation can be reduced using new cultivation technology and automation. Here, we test and demonstrate successful culture conditions and medium compositions for in vitro initiation, multiplication, and rooting of the Southern highbush cultivar ‘Blue Suede™’ (*Vaccinium* hybrid).

## 1. Introduction

Blueberry (*Vaccinium* L. sp.) is a globally utilized fruit crop with high health benefits due to its antioxidant and anti-inflammatory properties [1,2]. From a family of about 500 species, the Southern highbush blueberry ‘Blue Suede^TM^’ (*Vaccinium* hybrid) developed in 2008 by the University of Georgia, Athens, GA, USA, ripens early compared with the Rabbiteye varieties common in the Southeast of the USA [3]. Seed-based propagation of this cultivar is not workable due to the high heterozygosity rate [4]. Instead, vegetative propagation by stem cuttings is used. However, it is a slow method to generate new plants compared with in vitro micropropagation [5]. The development of effective methods for rapid propagation of superior genotypes throughout the year is crucial to capture the benefits of new blueberry cultivars as commercial crops and can be achieved using scalable in vitro methods based on bioreactors [6,7]. Most of the research on blueberry propagation focuses on highbush and lowbush cultivars [5,6,8,9,10,11,12,13,14,15,16,17,18,19,20,21,22,23]. However, the propagation processes are genotype-specific and each cultivar requires a tailored propagation protocol for optimal performance [24]. Suitable micropropagation media and methods for the blueberry ‘Blue Suede^TM^’ (*Vaccinium* hybrid) have not been previously studied. Here, we explore possibilities to utilize different culture vessel types to establish an effective propagation process from in vitro initiation, multiplication, and rooting for this commercially promising hybrid.

While semi-solid gelled medium is the most commonly used method in blueberry propagation [15], bioreactor systems with liquid medium have the potential for large-scale commercial production of micropropagated plants due to their ability to provide more control over the microenvironmental condition [25]. However, bioreactors based on full immersion in liquid medium can cause hyperhydricity, shear stress, and affect the growth and development of the culture negatively [26,27]. Temporary immersion bioreactors (TIBs) can be used to overcome these challenges by allowing for the immersion of the explants in liquid medium for limited intervals rather than permanent immersion [6,13,28,29,30]. TIBs have also been successfully tested with the lowbush blueberry (*Vaccinium angustifolium*) cultivar Fundy, ‘NB1′and ‘QB1′, showing that the shoot number per explant could be three times higher than that on semi-solid medium [17]. In the present study, the TIB model from BSI (Bioautomaton System Inc., Atlanta, GA, USA) was tested for the multiplication of ‘Blue Suede^TM^’ blueberry. This TIB model has an optimized design with separate medium and culture compartments and a choice for tissue support. It was previously shown to stimulate shoot multiplication rates in *Betula pendula*, *B. pubescens*, and *Eucalyptus grandis* × *Eucalyptus urophylla* [31], and to increase the proliferation rate of immature somatic embryos and the yield of mature somatic embryos of *Abies nordmanniana* [31], *Picea abies* [32], and *Larix* × *eurolepis* Henry [33].

The aim of this study was to develop an effective micropropagation process for the ‘Blue Suede^TM^’ (*Vaccinium* hybrid) blueberry. Various culture systems based on semi-solid and liquid culture mediums and different compositions of media were tested to support the increased utilization of this blueberry cultivar as a commercial crop.

## 2. Results

### 2.1. Shoot Culture Establishment of ‘Blue Suede^TM^’ Blueberry

**Figure 1:**
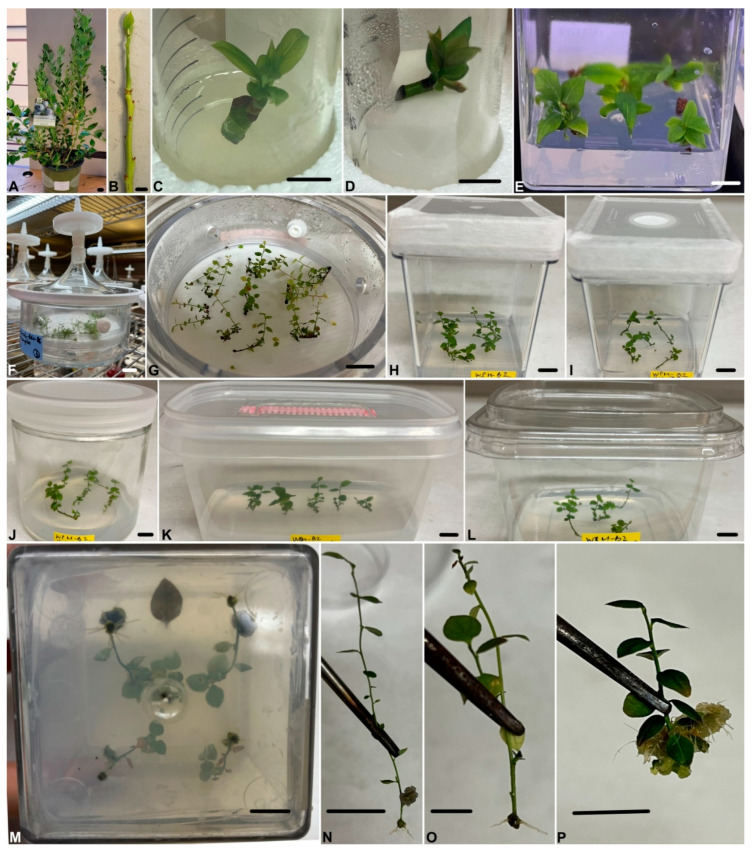
In vitro propagation of ‘Blue Suede^TM^’ blueberry. The starting material was collected from nursery stock plants (**A**). The stems were dissected into small segments which contained 1–2 axillary buds after defoliation (**B**). New shoots formed after 4 weeks on medium WPM-B2: on semi-solid medium in tubes (**C**), liquid medium in tubes (**D**), and semi-solid medium in magenta boxes (**E**), and in liquid medium in TIBs (**F**,**G**), magenta boxes with closure PTL-100C™ (**H**), magenta boxes with closure PTL-100C™ vented (**I**), glass jars (**J**), microbox containers Sac O2 (**K**), and SteriCon™-8 (**L**). Rooting occurred in vitro in magenta boxes on ½WPM-R2 medium (**M**,**N**), ½WPM-R1 medium (**O**), and ½MS-R1 medium (**P**). Scale bar: 1 cm.

**Figure 2:**
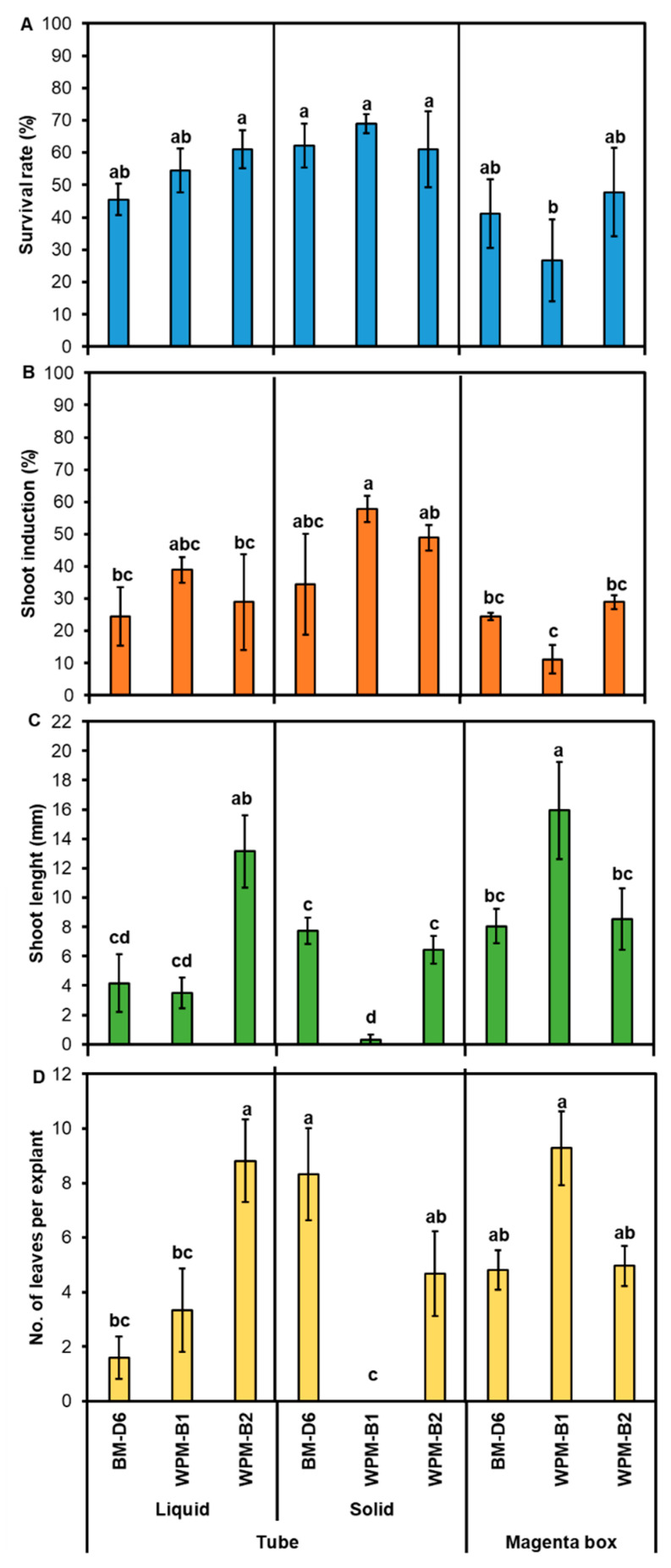
Effect of different culture systems and culture media on establishment and growth of ‘Blue Suede™’ (*Vaccinium* hybrid) in vitro cultures 4 weeks after in vitro initiation: (**A**) percentage survival rate, (**B**) shoot induction rate, (**C**) shoot length (mm), and (**D**) number of leaves per explant. Data represent mean ± SE from three replications. Different letters indicate significant differences at *p* < 0.05 according to Duncan’s multiple range test (*n* = 30).

To start in vitro cultures, nursery stock plants (Figure 1A) were used as starting material to collect nodal explants with 1–2 axillary buds (Figure 1B). The axillary buds showed sprouting in all culture systems and media after 3–4 weeks (Figure 1C–E). In the tube culture system, there was a slightly different survival rate for segments in liquid medium, with the highest survival rate observed in WPM-B2 medium (Figure 1D). There was no significant difference in the survival rate on solid medium (Figure 1C and Figure 2A). In the magenta box culture system, the segments cultured on WPM-B2 showed the lowest survival rate (Figure 2A), while explants on WPM-B1 and BM-D6 medium showed the same survival rate (Figure 1E and Figure 2A).

In general, shoot induction was higher on the WPM-B1 medium in tubes compared with magenta boxes both in liquid and on solid media (Figure 2B). The highest shoot induction rate of 57.8% was recorded when the segments were cultured on solid WPM-B1 medium in tubes (Figure 2B). By contrast, the lowest shoot induction rate at 11.1% was recorded on WPM-B1 medium in magenta boxes (Figure 2B).

A similar pattern was found for shoot length and number of leaves per explant, irrespective of culture medium or system (Figure 2C,D). The highest shoot length was observed in WPM-B2 liquid medium in tubes (13.15 ± 2.46%) and on WPM-B1 in magenta boxes (15.94 ± 3.31%) (Figure 2C). The shortest shoot length was recorded on WPM-B1 solid medium in tubes (Figure 2C). Furthermore, the highest number of leaves was observed on WPM-B1 medium in magenta boxes (9.28 ± 1.36), followed by WPM-B2 liquid medium in tubes (8.80 ± 1.52) and BM-D6 solid medium in tubes (8.32 ± 1.68), while the lowest number of leaves was observed on solid WPM-B1 medium in tubes (Figure 2D).

### 2.2. Multiplication of ‘Blue Suede^TM^’ Blueberry

**Figure 3:**
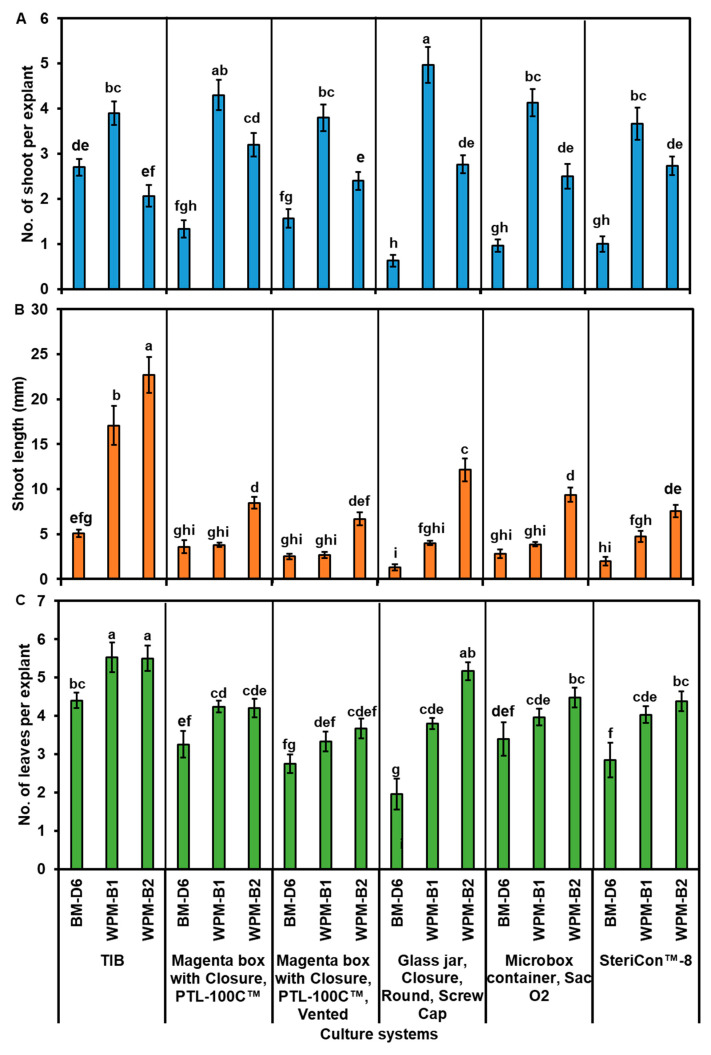
Effect of different culture systems and culture media on multiplication of established in vitro cultures of ‘Blue Suede™’ (*Vaccinium* hybrid) explants after 4 weeks of multiplication: (**A**) number of shoots per explant, (**B**) shoot length, and (**C**) number of leaves per explant. Data represent mean ± SE from three replications. Different letters indicate significant differences at *p* < 0.05 according to Duncan’s multiple range test (*n* = 30).

Growing shoots (1.5–2.0 cm) from the initial culture were selected to start multiplication experiments. All leaves were removed from the segments before being placed into three different media (BM-D6, WPM-B1, and WPM-B2) and six different culture systems: TIBs, magenta boxes with closure PTL-100C™, magenta boxes with closure PTL-100C™ vented, glass jars, microbox containers Sac O2, and SteriCon™-8. The results are shown in Figure 1G–L and Figure 3. Across culture systems, the highest shoot multiplication rate was observed with segments cultured on WPM-B1 medium, followed by WPM-B2, and the lowest on BM-D6 medium, except in TIBs, where the number of shoots was lowest on WPM-B2 medium (Figure 3A). The highest (4.97 ± 0.40) and the lowest (0.63 ± 0.13) number of shoots were recorded in glass jars (Figure 3A).

Although the segments cultured in TIBs did not show the highest number of shoot formations, the highest shoot lengths were recorded in this culture system on WPM-B1 and B2 medium (17.09 ± 2.15 mm and 22.71 ± 2.01 mm, respectively) (Figure 1G–L and Figure 3B). A similar pattern was observed in each culture system where the shoot lengths increased from BM-D6 to WPM-B1 and then WPM-B2 (Figure 3B).

The results in Figure 3C show that the number of leaves per explant was higher when the segments were cultured on WPM-B1 or B2 and lower on BM-D6 medium, regardless of the culture systems. The highest number of leaves was found in TIBs on WPM-B1 and B2 medium (5.53 ±0.38 and 5.50 ± 0.33, respectively) (Figure 3C). The segments cultured on BM-D6 medium in glass jars showed the lowest number of leaves (Figure 3C).

The scalability of ‘Blue Suede^TM^’ blueberry was tested by assessing the shoot induction rates after continuous culture of shoots during three subculture intervals (12 weeks). The shoot induction rates for continuous cultures were also highest for WPM-B1 (Table 1). There were no signs of decline of the continuous culture, indicating that the cultures could be continued for extended time periods to accumulate more shoots.

### 2.3. Rooting of ‘Blue Suede^TM^’ Blueberry

Shoots were cultured for four weeks in different media to induce root formation (Table 2). The results show significant differences in terms of rooting rate (%), root length (mm), and number of roots per explant depending on medium composition and plant-grown regulators (Table 2). The most successful rooting medium across the measured parameters was ½WPM-R2, composed of half-strength WPM basal medium supplemented with 0.5 mg·L^−1^ IBA and resulting in a rooting rate of 90.00 ± 5.77%, 4.20 ± 0.55 roots per explant and the longest roots of 3.23 ± 0.34 mm. The lowest rooting rates occurred on half-strength MS and WPM medium without PGRs. Moreover, the rooting rates, number of roots per explant, and the root length were found to decrease with increasing IBA concentration in the BM-D9-R1 medium base. Specifically, the rooting rate decreased from 10.00 ± 0.87% to 6.67 ± 0.88% and then to 0% with increasing IBA concentrations from 0.5 to 1.0 and 3.0 mg·L^−1^, respectively. On the other hand, when using the half-strength of WPM medium supplement with 0.1 to 0.5 mg·L^−1^ IBA, the rooting rate increased from 10.00 ± 1.15% to 90.00 ± 5.77%, the number of roots also increased from 1.10 ± 0.38 to 4.20 ± 0.55 roots per explant, and the root length also increased from 2.05 ± 0.50 to 3.23 ± 0.34 mm. Additionally, the results showed that 13.33 ± 1.67% of the shoots cultured on WPM medium supplemented with 2.0 mg·L^−1^ IBA formed roots. The rooting rate, number of roots, and root length were also found to decrease at lower concentrations of MS basal medium with lower NAA concentrations.

## 3. Discussion

Blueberries have been cultivated in the USA since 1961 [34]. Many studies have contributed to improving the uses of the species and different cultivars developed for increased crop quality and yields, as well as better propagation and cultivation methods [15], but there are, to date, only a few reports for ‘Blue Suede^TM^’ blueberry [3,35]. Further improvements to the propagation methods for ‘Blue Suede^TM^’ blueberry plant production, from in vitro micropropagation to field growth, will support the increased use of this productive cultivar. To the best of our knowledge, this is the first report on micropropagation using nodal segments from ‘Blue Suede^TM^’ blueberry and specifically exploring the application of temporary-immersion bioreactors for the scale-up of plant production.

The initial in vitro induction and establishment of blueberry can be demanding and time consuming, where the selection of explants and seasonal timing is essential. It is generally recommended to initiate explants during early summer when the new plant has hardened slightly but has yet not entered dormancy. However, due to the limited scope of the published studies focusing on a few cultivars and species, there is a need for more comprehensive information on the optimal conditions that support substantial initial shoot growth from the diverse range of germplasm collections [4]. Here, we investigated the effect of culture media and culture systems on inducing and establishing in vitro cultures of ‘Blue Suede^TM^’ blueberry. The results confirm that different culture media and culture systems can be used to optimize the shoot formation efficiency.

In blueberry, the optimum medium and growth regulators vary between cultivars [15,36]. Modified basal medium for cranberry (BM-D6) [37] and WPM medium have been successfully used for shoot induction in a number of different *Vaccinium* species [15]. BM-D6 contains more NH_4_NO_3_, (NH_4_)_2_SO_4_, Ca(NO_3_)_2_, MgSO_4_, NaH_2_PO_4_, and C_12_H_22_CaO_14_ (calcium gluconate) than WPM. The present study showed that WPM was more effective for in vitro shoot induction than BM-D6 in ‘Blue Suede^TM^’ blueberry (Figure 2). The most effective medium for initiating and establishing an in vitro culture and to obtain rapid shoot formation was WPM medium supplement with a high concentration of 2iP (5 mg·L^−1^), regardless of the culture system and medium condition (Figure 1C–E) [38,39].

To stimulate the growth of axillary buds and prevent the formation of callus, the explant was placed on a medium with limited amounts of auxins but high levels of cytokinins. The cytokinins serve to overcome apical dominance and stimulate the development of branches from the sides of the leaf axis [37]. 2iP concentrations between 0 and 30 mg·L^−1^ stimulated shoot formation, and the maximum number of shoots was obtained at 28.8 mg·L^−1^ in lowbush blueberry (*V. angustifolium*) [40], while three highbush blueberry clones (G-694, G-355, and G-224) induced fewer shoots at 30 mg·L^−1^ than at lower concentrations [41]. The best shoot multiplication of highbush blueberry (*Vaccinium corymbosum*) also occurred at 5 mg·L^−1^ 2iP [38]. When comparing 2iP and zeatin, zeatin at 2 mg·L^−1^ was most effective for shoot induction and, overall, was more effective than 2iP for highbush blueberry (*V. corymbosum*). No shoot proliferation occurred on medium without zeatin in this cultivar [42]. Zeatin alone has also previously been shown to stimulate shoot proliferation on *V. corymbosum* ‘Bringtwell’ [43]. In *V. corymbosum* ‘G-694′, ‘G-355′, and ‘G-224′, the addition of 2iP at 15 mg·L^−1^ resulted in an increase number of shoots, whereas no usable shoots longer than 1 cm were observed at 20 mg·L^−1^ 2iP in *V. corymbosum* ‘Bringtwell’ [41,43]. Similarly, zeatin was also reported as the best growth regulator for the in vitro establishment of *Vaccinium virgatum* ‘Delite’ [4]. In the Southern highbush blueberry variety ‘Blue Suede^TM^’, the combination of 2iP and zeatin was effective for shoot induction in tubes but not in magenta boxes (Figure 2). However, the shoots formed were tiny and lacked leaves (not shown). The shoots formed on medium supplement with only 2iP, however, grew tall and had more leaves (not shown).

The physical state of the medium (liquid or solid) also influenced the frequency of shoot initiation and growth. Almost all studies on shoot induction in blueberry use solidified medium [15]. Liquid culture medium offers several advantages over solidified medium. The use of gelling agents significantly contributes to the production costs of in vitro culture and can lead to issues related to impurities or inconsistent batch quality [44,45]. Different types of liquid micropropagation systems have been developed, including immersion systems where explants are continuously or periodically suspended in the medium, and other systems that use inert materials like filter paper (paper bridge) to support the explants [46,47,48,49]. The present study showed no significant difference between solid and liquid medium for shoot induction of ‘Blue Suede^TM^’ blueberry, but the shoot length was longer and the number of leaves per explant was higher in liquid than on solid medium during multiplication (Figure 2).

Furthermore, data show that the shoot multiplication capacity of TIBs are at least ten times higher than any of the semi-solid culture systems tested based on surface area (Table 1 and Table 2). The results also indicate that continuous cultures could be maintained for prolonged times without loss of growth by only replacing the liquid culture medium and not dividing the shoots. Therefore, using a TIB system for sustainable growth and multiplication could offer a more cost-effective method for scalable shoot multiplication.

Significant differences were observed between TIBs and solid medium after 4 weeks in different proliferation medium and culture systems, with the shoots grown in TIBs exhibiting the longest shoots, highest number of leaves, and biggest leaves (Figure 1G–L and Figure 3). In the TIBs, the explants were in closer contact with the media and had more space, which may explain the higher shoot proliferation, shoot length, and number of leaves compared with others culture systems. A TIB system was also found to be three times more efficient for in vitro propagation than solid medium for lowbush blueberry (*V. angustifolium*) [17]. TIBs are also promising as an alternative system for the mass propagation of raspberry and strawberries, as demonstrated by higher multiplication rates of 4.1 and 6.7 compared with 1.9 and 3.2 on solid medium [50]. For other blueberry cultivars, shoot proliferation was also higher in TIBs for highbush (*V. corymbosum* ‘Polaris’), half-high (*V. corymbosum* ‘St. Cloud’), and hybrid blueberries (clones ‘S1ʹ,‘S2ʹ, ‘S3ʹ, ‘S4ʹ, ‘S5ʹ, and ‘S6ʹ) [6].

Blueberries are today mostly propagated through regular cuttings and, to a lesser extent, through in vitro micropropagation. In vitro rooting with commonly used methods typically takes up to 8 weeks, with varying success rate varies depending on the cultivar. In *V. corymbosum,* different cultivars produced roots after 8 weeks with different success rates, ranging from 84 to 100% on ½WPM medium [12], 30 to 70% in ½WPM medium [51], and at a 28% success rate on ½ OM (Olive medium) [14]. The rooting success rate for ‘Blue Suede^TM^’ was evaluated after 4 weeks, and the highest rooting success rate was 90% on ½WPM. In the present study, we compared several medium compositions based on previous results. We found that there is an apparent benefit to rooting on half-strength basal medium compared with full-strength, as the two top rooting rates, 90% and 33%, respectively, arweree noted on ½ WPM and ½ MS (Table 2). Successful rooting can occur on full-strength culture medium, although it is common to reduce the basal medium concentration for the rooting of woody ornamentals, fruit trees, and forest tree species [52,53]. Furthermore, the results presented here also point to the importance of selecting the best PGR and using it at the optimal concentration, where specifically relatively higher auxin levels seem to be required for the rooting of ‘Blue Suede^TM^’ (Table 2).

## 4. Materials and Methods

### 4.1. Plant Materials, Sterilization Process, and Culture Initiation

Newly formed stems (10–15 cm in length) from vigorously growing plants were collected from nursery stock plants (Figure 1A). The stems were dissected into small segments which contained 1–2 axillary buds after defoliation (Figure 1B). The segments were rinsed with running tap water and adding 2–3 drops of Tween 20 (Sigma-Aldrich, Saint Louis, MO, USA) for 30 min. The rinsed segments were transferred to a laminar flow hood and soaked in 70% ethanol for 10 s, rinsed with sterile distilled water three times, then immersed for 8 min in 0.1% HgCl_2_ with agitation, and then rinsed again with sterile distilled water three more times. The surface sterilized segments were transferred to autoclaved filter paper to remove excess water and then placed on three different initiation media (Table S1): (1) BM-D6 medium [54] supplemented with 2 mg·L^−1^ of 6-(γ,γ-dimethylallylamino) purine (2-iP) (PhytoTech Labs, Lenexa, KS, USA) and 25 g·L^−1^ of sucrose (PhytoTech Labs, USA); (2) WPM medium [55] (PhytoTech Labs, USA) supplemented with 2 mg·L^−1^ zeatin (PhytoTech Labs, USA), 3 mg·L^−1^ 2-iP, and 30 g·L^−1^ of sucrose (WPM-B1) [56]; and (3) WPM medium supplemented with 0.1 g·L^−1^ of myo-inositol (Sigma, USA), 5 mg·L^−1^ 2-iP, and 20 g·L^−1^ of sucrose (WPM-B2) [57]. The pH was adjusted to 5.0 through the addition of HCL 1N or KOH 1N prior to sterilization at 121 °C, 1 atm for 20 min. All media were solidified by the addition of 6 g·L^−1^ of agar (PhytoTech Labs, USA). The segments were initiated in three different culture systems: (1) 50 mL glass tubes with 10 mL solid medium; (2) 50 mL glass tubes with 10 mL liquid medium and a filter-paper bridge; and (3) magenta boxes with closure PTL-100C™ with 50 mL medium. The segments were subcultured onto the same medium every 4 weeks for initial induction of shoots. After 8 weeks of culture, the survival rate (%), shoot induction rate (%), shoot length (mm), and number of leaves per shoot were recorded.

### 4.2. Proliferation Using Different Culture Systems and Medium

The initial in vitro shoots were carefully separated into uniformly sized individual segments that measured between 1.5 and 2 cm in length by removing all fully developed leaves. The culture systems was tested with a total of 30 segments, with ten segments used in each of three TIBs (Bioautomaton System Inc., Atlanta GA, USA) and five segments used in each of six of the following containers (Table 3): magenta box with closure PTL-100C™ (PhytoTech Labs, USA), magenta box with closure PTL-100C™ vented (PhytoTech Labs, USA), glass jar (C597, PhytoTech Labs, USA), microbox container Sac O2 (Nevele, Belgium), and SteriCon™-8 (PhytoTech Labs, USA). The same three medium, BM-D6, WPM-B1, and WPM-B2, used for initiation were also used in the proliferation experiments (Table S1). The number of shoots per initial explant, shoot length (mm), and number of leaves per shoot were investigated after four weeks of culture. The use of the TIBs has been described previously for micropropagation of birches and Eucalyptus [32]. Briefly, shoot segments were placed on a filter paper (Whatman 113, Sigma, USA) placed on the screen of the bioreactor. The cultures were temporarily immersed with culture medium one time per 24 h. The setting allowed the medium only to soak the tissue, then recede.

### 4.3. Rooting Using Different Media

Different compositions of rooting medium were tested (Table S1). Shoots (3–4 cm length) were rooted using magenta boxes in media M-D9-R1, 2, and 3 [55] supplemented with indole-3-butyric acid (IBA 0.5, 1.0, and 3.0 mg·L^−1^, respectively), WPM-R1 composed of WPM medium [55] (PhytoTech Labs, USA) supplemented with 2 mg·L^−1^ IBA (Sigma, USA), ½WPM-R1, and 2 [51], which was half-strength WPM medium supplemented with IBA (0.1 and 0.5 mg·L^−1^, respectively); MW-R1 [58] composed of half-strength MS medium (PhytoTech Labs, USA) [59] and half-strength WPM medium (PhytoTech Labs, USA) [55] without plant-growth regulators [58]; ½MS-R1 [60] composed of half-strength MS medium [59] supplemented with 2 mg·L^−1^ 1-Naphthaleneacetic acid (NAA, Chem Service Inc., West Chester, PA, USA) and ¼MS-R1, which was included with quarter-strength MS medium (PhytoTech Labs, USA) [59] supplemented with 0.1 mg·L^−1^ NAA. Percentage of rooted plants, number of roots, and root length (mm) were determined after 4 weeks of culture.

### 4.4. Growth Conditions

During the initial in vitro induction phase, proliferation, and rooting, all types of cultures were cultivated at a temperature of 23 ± 2 °C under a 16/8 h light–dark cycle with a light intensity of 40 µmol·m^−2^·s^−1^ produced by daylight fluorescent lamps (Aqueon, Franklin, WI, USA).

### 4.5. Statistical Analysis

All experiments were carried out with three replications with a total of 30 explants per treatment. The mean ± standard error (SE) was calculated. Statistical analysis was performed using one-way analysis of variance (ANOVA) followed by Duncan’s test, using the SPSS statistic 16.0 software (SPSS Inc., Chicago, IL, USA). Different letters indicate significant difference at *p* < 0.05. 

## 5. Conclusions

In conclusion, this study demonstrates the benefits of protocol testing and suggests useable protocols for in vitro initiation, multiplication, and rooting of ‘Blue Suede^TM^’ blueberry. Here, we show that shoots can be initiated in vitro in tubes using solid WPM medium supplemented with 2iP (5 mg·L^−1^), but that the TIBs then offer a more promising culture-vessel alternative for fast shoot-multiplication of ‘Blue Suede^TM^’ blueberry. The application of WPM medium supplemented with 2iP (3 mg·L^−1^) and zeatin (2 mg·L^−1^) is suitable for shoot multiplication, while WPM medium supplemented with 2iP (5 mg·L^−1^) can be used to promote shoot elongation. The best rooting results were obtained on half-strength basal media. This study is the first to demonstrate effective in vitro multiplication and rooting of ‘Blue Suede^TM^’ blueberry, and the results will support scaling-up plant production of ‘Blue Suede^TM^’ blueberry.

## Figures and Tables

**Table 1:** Test of scalability using TIBs for ‘Blue Suede^TM^’ blueberry shoot multiplication. Rate of shoot induction after three months of continuous TIB culture. Letters indicate significant differences at *p* < 0.05 according to Duncan’s multiple range test (*n* = 50).

Medium	Shoot Induction (%)
BM-D6	253.33 ± 73.76 b
WPM-B1	849.33 ± 53.05 a
WPM-B2	725.33 ± 43.23 a

**Table 2:** Effect of different media compositions on rooting of shoots of ‘Blue Suede^TM^’ blueberry after 4 weeks in gelled medium. Percentage of shoots forming roots, number of roots per explant, and average root length on each medium are presented. Data represent mean ± SE from three replications. Different letters indicate significant differences at *p* < 0.05 according to Duncan’s multiple range test (*n* = 30).

Medium	IBA (mg·L^−1^)	NAA (mg·L^−1^)	Rooting (%)	No. of Roots Per Explant	Root Length (mm)
BM-D9-R1	0.5	0	10.00 ± 0.87 cd	0.40 ± 0.31 cd	0.09 ± 0.08 d
BM-D9-R2	1	0	6.67 ± 0.88 cd	0.23 ± 0.18 cd	0.05 ± 0.03 d
BM-D9-R3	3	0	0.00 ± 0.00 d	0.00 ± 0.00 d	0.00 ± 0.00 d
MW-R1	0	0	0.00 ± 0.00 c	0.00 ± 0.00 d	0.00 ± 0.00 d
½WPM-R1	0.1	0	10.00 ± 1.15 cd	1.10 ± 0.38 bc	2.05 ± 0.50 b
½WPM-R2	0.5	0	90.00 ± 5.77 a	4.20 ± 0.55 a	3.23 ± 0.34 a
WPM-R1	2	0	13.33 ± 1.67 c	0.17 ± 0.08 d	0.08 ± 0.05 d
½MS-R1	0	2	33.33 ± 8.82 b	1.37 ± 0.48 b	0.81 ± 0.23 c
¼MS-R1	0	0.1	3.33 ± 0.60 cd	0.03 ± 0.03 d	0.00 ± 0.00 d

**Table 3:** Description of the selection of culture vessels tested for shoot multiplication. Standard medium volume, culture surface area, and reusability.

Culture System	Culture System’s Volume (mL)	Culture System’s Surface Area (cm^2^)	Reusable	Medium Volume (mL)
TIB	880	132.73	Yes	500
Magenta box with Closure, PTL-100C™	372	56.25	Yes	50
Magenta box with Closure, PTL-100C™, Vented	372	56.25	Yes	50
Glass jar, Closure, Round, Screw Cap	473	62.21	Yes	70
Microbox container, Sac O2	540	81.25	7 times	80
SteriCon™-8	473	103.23	No	70

## Data Availability

Data sharing not applicable. No new data were created or analyzed in this study. Data sharing is not applicable to this article.

## Supplementary Materials

The following supporting information can be downloaded at: https://www.mdpi.com/article/10.3390/plants12152752/s1, Table S1: Medium compositions used for ‘Blue SuedeTM’ blueberry in vitro initiation, multiplication, and rooting.
