# Supplementary material for: In Vitro Propagation of the Blueberry ‘Blue Suede™’ (Vaccinium hybrid) in Semi-Solid Medium and Temporary Immersion Bioreactors
Source: Plants (Basel). 2023 Jul 25;12(15):2752. doi: 10.3390/plants12152752 (PMC10421453; doi:10.3390/plants12152752)
Supplement: Supplementary file 1 [file plants-12-02752-s001.zip › plants-2481766-SI.pdf]

Supplementary Table S1: Medium compositions used for ‘Blue SuedeTM’ blueberry in vitro initiation, multiplication and rooting.

Mineral	Component (mg/L)	WPM-B1&B2	BM-D6	BM-D9-R1,2,3	MW-R1	1/2WPM-R1,2	MS-R1	1/2MS-R1	1/4MS-R1
Macro elements	NH4NO3	400.00	550.00	550.00	1025.00	300.00	1650.00	825.00	412.50
	(NH4)2 SO4	-	50.00	50.00	-	-	-	-	-
	CaCl2	72.50	-	-	202.35	36.25	332.20	166.10	83.05
	Ca(NO3)2	386.00	410.00	410.00	193.00	193.00	-	-	-
	MgSO4.7H2O	180.70	370.00	370.00	180.70	90.35	180.70	90.35	45.18
	KCl	-	-	-	-	-	1900.00	950.00	475.00
	KNO3	-	330.00	330.00	950.00	-	170.00	85.00	42.50
	KH2PO4	170.00	220.00	220.00	170.00	85.00	-	-	-
	K2SO4	990.00	140.00	140.00	495.00	495.00	-	-	-
	NaH2PO4	-	100.00	100.00	-	-	-	-	-
	Calcium gluconate	-	1300.00	1300.00	-	-	-	-	-
Micro elements	Na2-EDTA	37.30	37.30	37.30	37.30	18.65	37.30	18.65	9.33
	FeSO4.7H2O	27.85	-	-	27.85	13.93	27.85	13.93	6.96
	H3BO3	6.20	6.20	6.20	6.20	3.10	6.20	3.10	1.55
	CoCl2.6H2O	-	0.03	0.03	0.01	-	0.03	0.01	0.01
	CuSO4.5H2O	0.25	0.03	0.03	0.14	0.13	0.03	0.01	0.01
	MnSO4.H2O	22.30	22.30	22.30	19.60	11.15	16.90	8.45	4.23
	Na2MoO4.2H2O	0.25	0.25	0.25	0.25	0.13	0.25	0.13	0.06
	KI	-	0.83	0.83	0.42		0.83	0.42	0.21
	ZnSO4.7H2O	8.60	8.60	8.60	8.60	4.30	8.60	4.30	2.15
Vitamins	Glycine	2.00	1.00	1.00	2.00	2.00	2.00	1.00	0.50
	Adenine hemisulphate	-	6.83	6.83	-	-	-	-	-
	Casein hydrolysate	-	40.00	40.00	-	-	-	-	-
	Myo-inositol	100.00	100.00	100.00	100.00	100.00	100.00	50.00	25.00
	Nictotinic acid	0.50	0.40	0.40	0.50	0.50	0.50	0.25	0.13
	Pyridoxine HCl	0.50	0.40	0.40	0.50	0.50	0.50	0.25	0.13
	Thiamine HCl	0.10	0.60	0.60	0.10	0.10	0.10	0.05	0.03
